# Imaging local soil kinematics during the first days of maize root growth in sand

**DOI:** 10.1038/s41598-021-01056-1

**Published:** 2021-11-15

**Authors:** Floriana Anselmucci, Edward Andò, Gioacchino Viggiani, Nicolas Lenoir, Chloé Arson, Luc Sibille

**Affiliations:** 1 Univ. Grenoble Alpes, CNRS, Grenoble INP, 3SR, 38000 Grenoble, France; 2 School of Civil and Environmental Engineering, Georgia Institute of Technology, Atlanta, GA USA

**Keywords:** Civil engineering, Biological physics, Imaging techniques

## Abstract

Maize seedlings are grown in Hostun sand with two different gradings and two different densities. The root-soil system is imaged daily for the first 8 days of plant growth with X-ray computed tomography. Segmentation, skeletonisation and digital image correlation techniques are used to analyse the evolution of the root system architecture, the displacement fields and the local strain fields due to plant growth in the soil. It is found that root thickness and root length density do not depend on the initial soil configuration. However, the depth of the root tip is strongly influenced by the initial soil density, and the number of laterals is impacted by grain size, which controls pore size, capillary rise and thus root access to water. Consequently, shorter root axes are observed in denser sand and fewer second order roots are observed in coarser sands. In all soil configurations tested, root growth induces shear strain in the soil around the root system, and locally, in the vicinity of the first order roots axis. Root-induced shear is accompanied by dilative volumetric strain close to the root body. Further away, the soil experiences dilation in denser sand and compaction in looser sand. These results suggest that the increase of porosity close to the roots can be caused by a mix of shear strain and steric exclusion.

## Introduction

Root-soil interaction is a strongly coupled phenomenon between a growing plant and soil. This interaction occurs in a portion of the environment with complex boundaries, which is referred to as the *rhizosphere*. Depending on the considered activity (root growth, exudation, and water uptake) the radial extension of the rhizosphere can range from sub-μm to supra-cm scales^1^. Hinsinger et al.^1^ defined the rhizosphere as one of the most bio-diverse ecological systems on Earth. On the one hand, root secretions, together with bacteria, fungi, protozoa, and viruses, alter soil properties such as the capacity of soil to retain water^2^, or soil microstructure by binding particles together^3,4^. On the other hand, soil conditions (e.g., density and water content) affect the growth rate and tropic responses of roots^5,6^. The overarching goal of this work is to contribute to the new field of bio-inspired geotechnics^7^ by understanding the best conditions to grow a healthy root system (thus, a plant) while enhancing soil properties.

Here, the focus is on the local kinematics of an inert soil induced by the plant root growth, i.e., biotic aspects are discarded. An earlier study by Dexter^8^ suggested a compaction of the soil around the growing root, decreasing exponentially with the distance from the root. The model presented by Dexter is only in partial agreement with some more recent findings, such as the ones by Helliwell et al.^9^, Koebernick et al.^10^ and Lucas et al.^11^. These authors observed a small zone of increased soil porosity close to the root, generally attributed to a geometric effect due to the steric exclusion of soil particles near the root surface. Further from the root (measurements are usually performed up to hundreds of μm or even 1–2 mm from the root), no systematic trend was observed and both densification or porosity increase of the soil have been measured, depending on the soil nature and initial state. Hence, the interpretation of the local modifications of soil porosity in these conditions is still an open question.

In this paper, maize plants (*Zea Mays L.*) are grown in Hostun sand and 3D images of root-soil systems obtained by X-ray computed tomography are analysed to measure the porosity and the displacement fields in the soil located up to few cm from the root surface. The germinated seedlings are scanned in-vivo for 8 days. Two different gradings of Hostun sand and two different initial bulk densities are used in order to produce different initial soil configurations and investigate the relation of the latter with the soil response to the root growth. Local strain tensors are computed from the displacement fields and characterised with their first and second invariants (representative of the volumetric and deviatoric deformations, respectively).

## Materials and methods

### Plant root system and granular soil

A wild type of *Zea Maize L.* is chosen for this study because its characteristics facilitate the observation of root-soil interactions under the constraints of in-vivo X-ray computed tomography—i.e., in a reduced soil volume (a few cm^3^) and during a rather short period (a few days). Maize produces a fibrous root system^12^ consisting in a group of roots with similar cross-section size and length. Roots do not penetrate deeply into the soil but rather create a thick network, which helps to hold soil particles together. According to the literature^13^, principal components of the maize root system develop within few days, with a growth rate of 1–4 cm/day and a mean root cross-section size ($\phi _R$) of 0.4–0.9 mm. In this study, root systems are generated from maize seeds placed in soil samples after germination, in order to control the initial shoot direction.

The soil chosen is sand, due to its coarse micro-structure (with respect to a clayey or silty soil) that facilitates phase segmentation in the image processing: the root system, soil particles, pores and pore water can be more clearly distinguished from each other. Hostun sand is considered here because its properties are well documented in the soil mechanics literature^14,15^. Hostun sand is extracted from a French quarry and is composed of sub-angular to angular grains made of 98% silica SiO_2_ and 2% metallic oxides. The material is extracted in clumps of different grain sizes. Two grain size distributions of Hostun sand are used in the present work: a finer size distribution, referred to as “HN31”, with a median particle diameter D_50_ of 338 μm, and a porosity *n* ranging from 0.39 to 0.51^16^, and a coarser size distribution, referred to as “HN1.5-2”, with a D_50_ of 1.9 mm and porosity ranging from 0.28 to 0.39^17^.

### Root-soil specimen set-up and image acquisition

**Figure 1:**
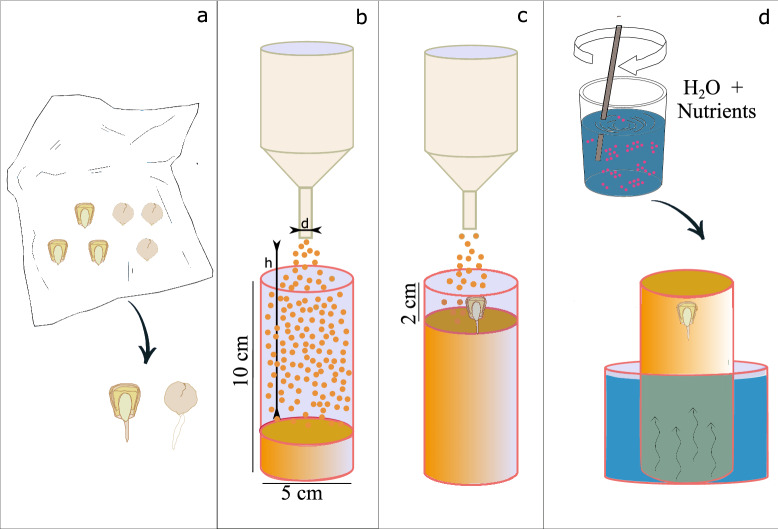
Main steps of the preparation of the root-soil specimens: (**a**) foil-wrapped seeds on germination filter paper, (**b**) sand deposition at controlled bulk density by air pluviation, (**c**) positioning of the germinated seed in the cell during sand pluviation, (**d**) watering of specimens using water enriched of nutrients.

Root-soil specimens are obtained by growing of a maize plant in a sand sample built in a cylindrical cell with an inner diameter of 5 cm and a height of 10 cm. Cell diameter was chosen as a trade-off between the field of view during the X-ray scanning and the spatial resolution. With the device adopted here, the voxel is equal to 40 μm. The cell is made of a polymethylmethacrylate (PMMA) tube sealed at the bottom with an *ad-hoc* 3D printed base meshed with holes to allow watering from the bottom. The specimen is built in four main stages, as illustrated in Fig. 1:

1. the maize seed is germinated and then sowed in sand. Each seed is sterilised in a solution composed of 15% commercial bleach and 85% distilled water for 15 min, then rinsed in distilled water 3 times, and eventually soaked in water for 15 min. Seeds are transferred in culture dishes on a 1.5 mm thick germination filter paper, foil-wrapped, and placed vertically in the dark for 48–72 h at a fixed temperature of 19 ± 2 °C;
2. after seed germination, sand deposition is conducted by dry pluviation to control the sand bulk density and to create a soil microstructure similar to that of natural sand deposits^18^. Coarse and fine sands are deposited following the same procedure. Pluviation factors depend on the grain size (i.e., funnel opening width – *d*) and on the desired bulk density (i.e., drop height – *h*, and pouring rate). Fine sand (HN31) is packed with two different densities, a looser state with a relative density (i.e., dimentionless parameter defining the compactness of the specimen) $D_R=28\%$ and a denser one with $D_R=79\%$. Coarse sand (HN1.5-2) is packed to a looser initial state, with same relative density to the looser state of the fine sand. A dense state of the coarse sand was not reachable due to constraints relative to the size of the cell and the particle size;
3. the germinated seed is placed in the sand sample, during the sand pluviation, at a depth of about twice the seed height (here, depth is calculated with reference to the free surface after pluviation);
4. finally, specimens are watered from the bottom, with nutrient-enriched water. No additional water is added during the 8 days of the experimental campaign.

Plant specimens are kept in a growth chamber under controlled temperature (19 ± 2 °C) and humidity (47 ± 4%). Each specimen is imaged using the X-ray computed tomography facility installed in Laboratoire 3SR (Grenoble, France) every 24 h ± 15 min for 8 days. Day 0 is defined as the day when the germinated seed is planted in the sand. Day 7 is the last day when the root system is imaged. Specimens are placed in the X-ray scanner for the time of the image acquisition only. After each scan they are returned to the growth chamber. 3D images have a voxel size of 40 μm, requiring a scan duration of about 2 h. Due to the unpredictable growth of the germinated seeds, three specimens per configuration are initially scanned. On Day 3, one specimen per configuration is chosen and analysed for the rest of the campaign. The specimen is chosen according to the orientation and length of the primary root. Supplementary Table S7 details the scan settings used during the experimental campaign.

To assess the possible effects of the X-rays on root growth, we conducted 3 reference experiments for each initial soil configuration outside of the CT scanner, with the same plant species as those imaged in the CT scan. In average, the leaf length, number of leaves and stem length were similar for the X-rayed and non-X-rayed plants grown in the same soil configuration. Furthermore, 10 days after the last X-ray scan, all the root systems were extracted and they exhibited primary roots of similar length and a similar number of first-order roots. Supplementary Table S8 presents a summary of the plant physical parameters when grown in and out of the CT scan. We did not observe any short-term X-ray effect on the development of the plants. The only noticeable difference among the specimens was soil water content: in looser fine sand, a higher level of evapotranspiration was observed in the X-ray scanned samples compared to those that were not scanned. This may be due to the temperature gradient between the growth chamber and the tomographer cabin.

### Image segmentation

The aim of the image segmentation is to produce, from each 3D greyscale image of the root-soil system, a four-phased volume where each voxel of the volume is attributed uniquely to one of the four phases (solid sand grains, root body, pore water, and pore air). Greyvalue thresholding is a basic way to segment images when phases consist of materials with clearly different densities, represented by different ranges of grey values in the image. This works here for the sand grains, which are clearly denser than the others components of the system, but cannot be applied for the segmentation of the roots, since their density is similar to the density of the pore water. A specific segmentation method has been developed for the root identification. Some image pre-processing steps are performed prior to the segmentation, and they are described in the following.

#### Pre-processing

For the set of images obtained for one given root-soil specimen, representative greyvalues attributed to each phase have to be comparable over all the images acquired in space (for every slice) and time (for every day), in order to ensure reliable quantitative phase assessments and effective measurements of the kinematics of the sand phase. Typically, the grey values of the peaks of the greylevel distributions are not expected to change over a set of images. Consequently, greylevel distributions are corrected by performing a linear contrast stretch of the images: the grey value of each voxel is scaled linearly with respect to the grey values at the peaks, such that they match for all the images (examples of grey level distributions before and after the linear contrast stretching are displayed in the Supplementary Table S9). In a second step, images are down-scaled by a factor of 2 to reduce the volume of data by a factor of 8, which has an added benefit of denoising the image. The pixel size is doubled to 80  μm. Noise reduction is further reduced by using a bilateral filter^19^. For further details and a comprehensive description of the image pre-processing, the reader is referred to Anselmucci’s thesis^20^.

#### Sand segmentation

The greyvalue threshold for the sand segmentation is determined once for each test from the image at Day 0. It is chosen such that the sand volume identified in the 3D image matches the volume deduced from the mass of dry sand introduced in the cell by pluviation. The analysis is performed one step further for the coarser sand (HN1.5-2). Since a grain diameter is about 38 pixels in the downscaled image, individual grains can be detected. This is achieved using a watershed algorithm^21^.

#### Root segmentation

**Figure 2:**
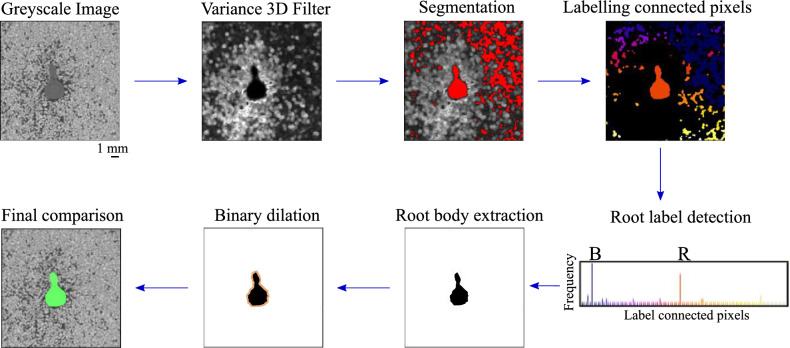
Steps of the image processing for the segmentation of the root system body from the greyscale image of the root-soil specimen. A 3D variance filter is applied to the original greyscale image, the output is segmented and each connected pixel is labelled. The frequency distribution of the new labelled image presents two main peaks corresponding to the background (B) and the root system (R). The segmentation is carried out in 3D, but a 2D cross section is displayed here for the sake of readability.

For root body segmentation, a method based on the edge identification using the 3D variance filter implemented in Image-J^22^ is proposed. The main steps are summarised in Fig. 2. This filter consists in replacing each voxel by the variance of the voxels contained in the 3D matrix centred in the voxel under investigation, within a distance $r_v$. Consequently, edges of phases with substantially different greylevel with respect to the neighborhood are highlighted. Typically, such a filter is not a suitable tool to segment sand grains in contact, which have a similar grey level. The value of $r_v$ has to be high enough to calculate a statistically representative average of the grey value of different phases (here, solid grain, water, and air) that are in the neighbourhood of the phase to detect (here, the root). In this study, $r_v=4$ pixels for fine sand and is set to $r_v=2$ pixels for the coarse sand. The radius is smaller for coarser sand because the contact among grains is smaller due to the bigger voids. As a result, the grey value of voxels representing root elements and big pores (filled with water or air), as shown in the red zones in Fig. 2, is clearly differentiated from that of voxels located at the interface between phases (light grey zones in Fig. 2). Thus, this technique helps to detect the edges of the interfaces among different phases, and root edges can be detected since the surrounding has a different mean grey scale value.

**Figure 3:**
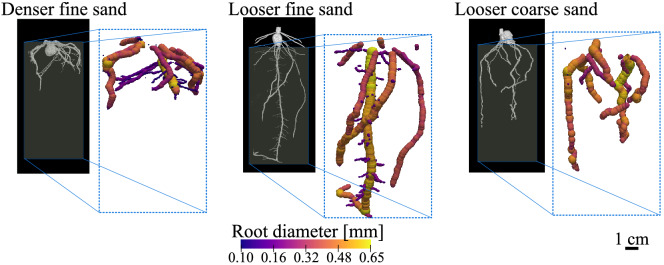
3D Maize root systems at Day 7 for each soil configuration; the colour-scale figures present the respective distributions of the root cross-section size.

The grey value of the root in the new image (i.e., post 3D variance filter) and big pores is then used for thresholding, and the volume is binarised according to that threshold as per Stamati^23^. At this stage, root body and big pores can still not be separated. To proceed, connected voxels of the same phase are clustered. Each cluster is labelled according to its volume. As shown in the frequency distribution of Fig. 2, the largest volume is identified as the image background (B), the second largest is attributed to the root body (R), and the other volumes are labelled as connected pores. At this stage, the identified root body is still incomplete because the root edge has been excluded from the thresholding, due to the variance filter. The full root body is recovered by dilating the selection of voxels representing the root *n* times, where *n* is half of the radius $\textit{r}_{v}$ used for the variance filter. The binarised identification of the full root body is compared to the initial greyscale image in the last step of Fig. 2 and displayed in 3D in Fig. 3. The proposed method makes it possible to precisely identify all the root elements with a cross-section size above about 4 times the pixel size (i.e., above 320 μm). Hence, with the pixel size considered here, the finer second and third orders lateral roots are not detected.

#### Water segmentation and reconstruction of the four-phased volume

Once sand particles and root system elements are identified, water and air phases can be segmented. The remaining voxels not assigned to root or sand are separated by a threshold value. Similarly to the sand segmentation, the threshold is chosen such that the volume of water identified from the image at Day 0 matches the water volume initially introduced in the sample.

**Figure 4:**
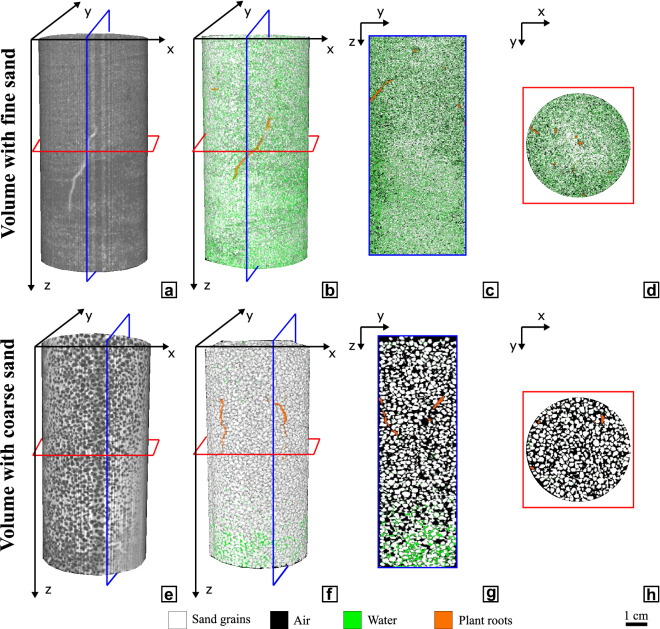
Phase segmentation of two root-soil specimens with the looser sand state – top row: fine Hostun sand (HN31), bottom row: coarse Hostun sand (HN1.5-2). (**a**, **e**) Greyscale volumes; (**b**, **f**) four-phased segmented volumes; (**c**, **g**) vertical section; (**d**, **h**) horizontal section.

In the case of the fine sand, pore sizes and more particularly the size of zones filled with water when the sand is partially saturated (i.e., pores are only partially filled by water) may be close to the voxel size. Therefore, most of the voxels attributed to soil pores can include both air and water phases and cannot be attributed rigorously to one of the two phases only. Consequently, water phase identification is only qualitative at the pore scale in the fine sand. In the coarse sand, pores are larger and water phase identification is more representative of the local water distribution. Figure 4 shows examples of reconstructed four-phase volumes.

### Statement on guidelines

Experimental research on cultivated maize plants and provision of maize seed comply with relevant institutional, national, and international guidelines and legislation.

## Results and discussion

### Root penetration and access to water depends on soil density

Parameters of the root system architecture are extracted from the voxelised root phase identified above. The first step consists in the 3D skeletonization of the root system with the python module skimage.morphology.skeletonise_3D^24^ – Supplementary Figure S1 provides an example of root skeleton. Then, an in-house developed algorithm identifies and labels each hierarchical root element, determines their respective length and calculates the size of the root cross-section at each voxel that makes up the root skeleton.

At this early stage of growth, the root size is independent from the soil conditions considered here, with a size of the primary root about 0.6 mm for all the root systems (Fig. 3). This value is in agreement with published data that report a mean size usually ranging between 0.4 and 1.0 mm for the maize primary root^13,25^. The root length density (RLD) is not correlated to the soil conditions either, with a RLD around 3000 m/m^−3^ at Day 7 in all the configurations (the time evolution of the RLD can be found in Supplementary Figure S3). By contrast, as displayed in Fig. 3, the sand density clearly affects the ability of the roots to penetrate deeply into the sand^6,26^. For the looser finer sand (HN31), the root system reached the bottom of the cell (at a depth of 100 mm below the sand surface) before Day 3, whereas the roots stopped at a depth of around 44 mm at Day 3 and were not able to grow deeper even after 7 days in the denser HN31 (the time series of the root depth is shown in Supplementary Figure S4). The effect of the soil configuration on the root growth can be also seen via the rates of elongation of the primary root (displayed in Supplementary Figure S5) which are mostly higher when the soil density is lower.

The higher resistance to penetration in denser sand can be interpreted as an effect of the sand macroscopic friction angle, which is higher at higher density. According to three drained triaxial compression tests performed under a very low confining pressure of 5 kPa (to represent the stress state of the shallow sand layers) on Hostun sand of various densities, the friction angle is 46.8° for the denser state and 37.7° for the looser one^27^. Our results show that the threshold of sand friction angle for maize root penetration in a fine granular soil is between these two limits, at least for the boundary conditions considered in the experiments. Concerning the coarse sand (HN1.5–2) in the looser state, root penetration is slower than in the looser fine sand, and the root reaches a depth of 87 mm at Day 7.

The root system growing in the looser fine sand is the one developing the greater number of laterals (from the primary, seminal, and crown roots) with more than 83 lateral roots at Day 7, whereas the number of lateral roots for the systems in the denser fine sand is 24 and that in the looser coarse sand is 14 (Supplementary Figure S2 provides the complete time series of the number of lateral roots recorded in each soil configuration for the single specimen observed). The spatial distribution of lateral roots is correlated to the map of degree of saturation (defined as the proportion of void space occupied by water and calculated from the distribution of the water phase in the four-phased volume). This observation confirms that soil water content in the vicinity of the root system has a strong influence on the development of lateral roots^28^. In coarser sand, the pores are larger, the capillary rise is thus lower, pore water is concentrated at the very bottom of the sand sample and roots do not reach such depth, even after 8 days. In the denser fine sand, pore water is initially homogeneously distributed. Nevertheless, roots fail to penetrate the soil and take up water present at the top of the specimen, which gets depleted in water, leaving no possibility for the roots to reach pore water. In looser fine soil, pore water is initially homogeneously distributed in the sand sample and the root system deploys in the whole wet sand volume.

Consequently, the different soil configurations considered in this study clearly impact the development of the root systems. The influence of bulk density dominates that of the mean sand grain size (at least for the range of grain sizes investigated), and resulting root systems are conform to what is commonly expected in these conditions^13,29^.

### Porosity change near the root depends on initial soil configuration

In the following, root development is considered as a loading applied to the soil, and the remainder of this paper is devoted to the study of the mechanical response of the soil to such a loading. Sand porosity is directly computed as the ratio of air and water filled pores volume to total soil volume from so-called trinarised 3D images.

**Figure 5:**
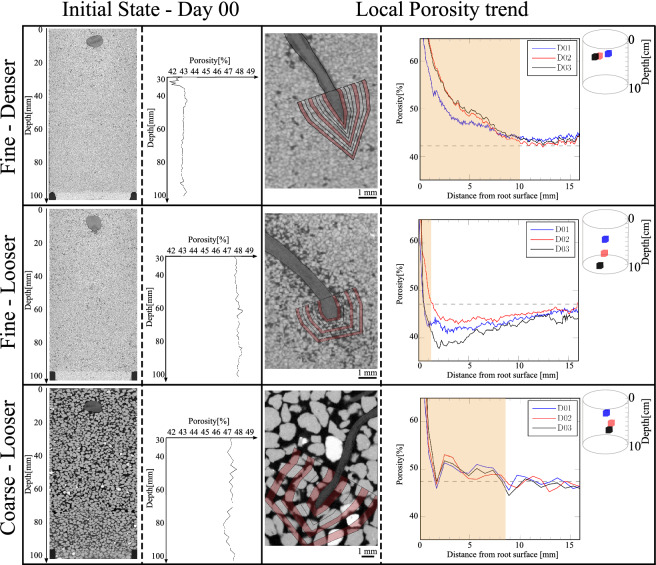
Vertical cross section of the greyscale image and vertical profile of sand bulk porosity at the initial state, Day 0 (left); change of the bulk porosity of sand with the distance from the surface of the primary root (the yellow area represents the distance range where the porosity is higher than the initial global one): for a given distance the porosity is computed within a hollow volume such as one of the red volumes drawn on the greyscale image around the root tip (center). The position of the small volume of soil used for the analysis is shown in the top right corner of each row.

The initial states of the samples are displayed in Fig. 5 with a vertical 2D slice (crossing the seed) of the greyscale volumes at Day 0 and the mean vertical porosity profiles computed on these 3D volumes. Porosity above the seed is not considered, as the presence of the seed at the end of sand pluviation may affect the initial porosity of the soil-root system. Fluctuations in the porosity profiles are not higher than ± 0.5% (except for the very top part of the finer denser sand sample) with a scattering slightly more pronounced in the coarser sand, directly induced by the bigger sand grains. In all cases, these results show the good homogeneity of the initial sand-root specimens. Furthermore, the initial porosity is clearly different for the finer denser sand ($\approx 43\%$) with respect to both looser sands, finer and coarser ($\approx 47.5\%$).

**Figure 6:**
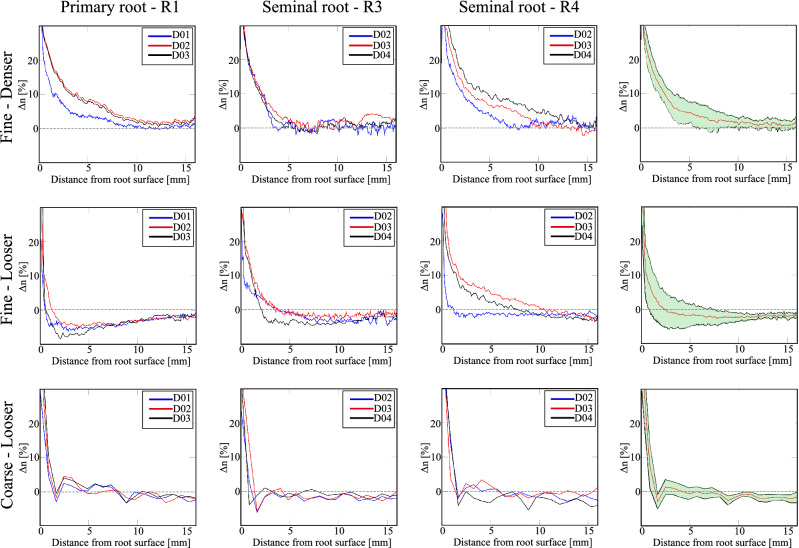
Variation of soil porosity $\Delta n$ with the distance from the root surface. Compilation of the results for three different root tips in the three different soil configurations. The last column shows the mean porosity along with the standard deviation (as a shaded area).

The local porosity distribution around the primary roots presented in Fig. 5 is computed over the hollow soil volume centered at the root tips and coaxial with the root axes. These hollow volumes, defined by dilating the external edge of the root body, have a thickness of $D_{50}/2$ (i.e., 160 and 800 μm for HN31 and HN1.5-2, respectively)^20^. The distance from the root surface (and from the root tip) ranges from 0 to more than 16 mm. The 16 mm distance was chosen because it was noted that porosity did not change beyond that distance. Porosity profiles can be drawn at different times (i.e., at different stages of the root growth). However, they were not further calculated three days after the shooting day of the considered root because other elements affecting the sand porosity (sprout of lateral roots or vicinity of the container edge to the root tip) interfered with the root body. It is worth noting that the range of variation of the porosity with the distance from the root is well above the fluctuations observed in the initial state and is thus representative of the alteration induced by the root growth.

In the same way, Fig. 6 compiles the profiles of the change of soil porosity $\Delta n$ (calculated as the difference between the porosity of the subvolume of interest at day *i* and that at day 0) for three root tips (the primary root and two seminal roots), and for every soil condition. The mean porosity profiles and the standard deviation from the mean are also presented in the last column of Fig. 6.

A significant increase of porosity in the direct root vicinity is noted regardless of the initial soil state. Nevertheless, the distance range over which such a porosity increase occurs depends on the initial soil state. The profiles of mean porosity indicate that the distance range is relatively large in the case of the fine denser sand, reaching a distance of about 10 mm (more than $30.0\,\times \text {D}_{50}$) from the root surface, whereas the increase of porosity is limited to about 2.5 mm ($\approx 7.4\,\times \text {D}_{50}$) in the fine looser sand. For the coarse looser conditions, the zone of increased porosity reaches a distance around 1.8 mm ($\approx 1.0\,\times \text {D}_{50}$). Such porosity increases near the root are in agreement with recent 2D observations performed with clayey or silty soils^9–11^. In previous studies^10^, porosity increase was attributed to steric exclusion, which depends on sand packing and the interface between the root and the grains. Steric exclusion could also explain observations made here in the looser sands, since it concerns few particles layers from the root surface^30,31^. For the coarse looser sand, this steric exclusion, involving almost a single layer of particles, is very limited. This is probably because roots are more prone to use the existing large pores to penetrate soil, thus limiting the movement of particles. In the denser sand, the porosity increase involves too many particle layers to be the consequence of steric exclusion only.

Further from the root surface, the porosity change is also affected by the initial packing porosity in the finer sand. For the denser case, porosity stabilises to a value slightly higher than, or close to, the initial bulk porosity; whereas for the looser sand, porosity is lower than the initial bulk one beyond a distance of 2.5 mm from the root surface. In other words, in denser fine sand, only dilation is observed, from the root surface until the maximum considered distance (16 mm), whereas in looser fine sand, dilation is observed near the root surface while compaction is noted further away. Consequently, the volumetric deformation of the soil in response to root elongation depends on the initial soil density. The case of the coarse sand in the looser state can be considered as an intermediate case between the two soil responses described above for fine sand: further from the root, porosity stabilizes close to the the initial bulk porosity with a slight tendency to compaction only.

Finally, the global spatial range over which sand porosity is affected (up to a distance of 10–15 mm) by the root growth is wider than what is usually reported in the literature, typically 1 mm in the rhizosphere from the root surface. This difference can be explained by the fact that the grain size used in this study is larger than the one usually considered in previous studies (e.g., Worcester clay loam soil investigated by Helliwell et al.^9^).

### Root elongation induces soil shearing and volumetric response

**Figure 7:**
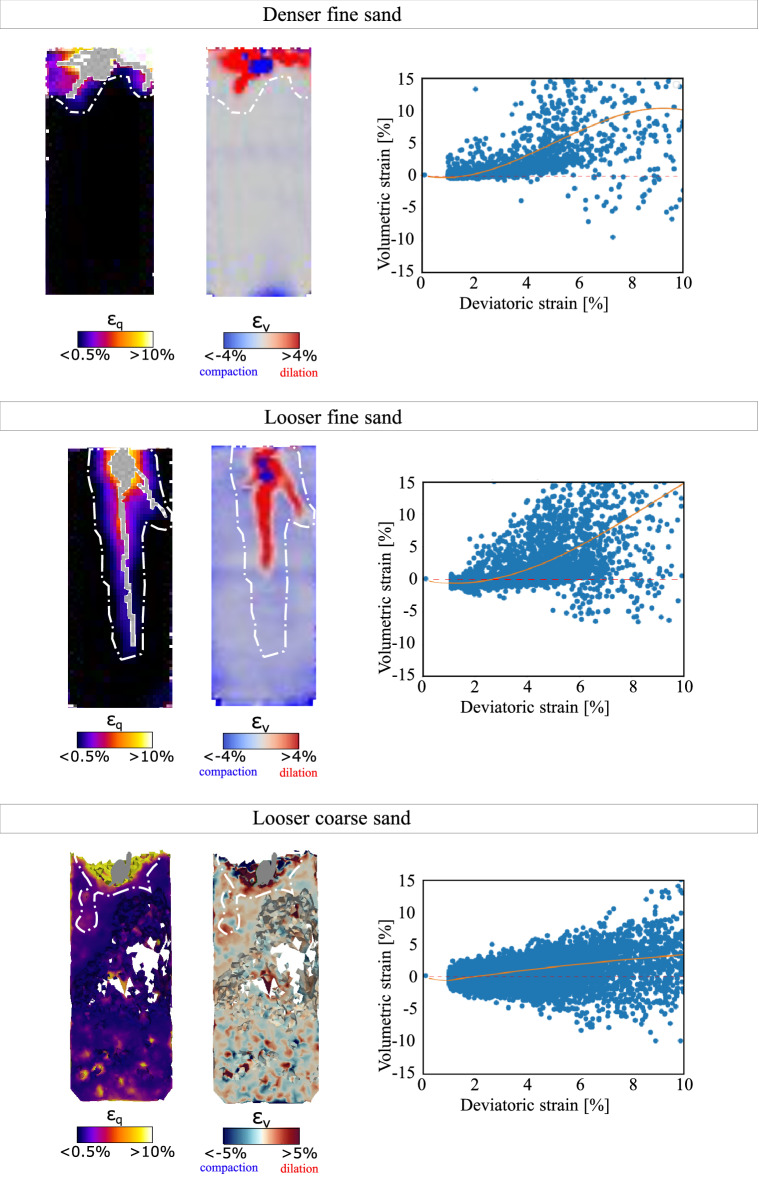
Identification of root-sheared soil with $\varepsilon _\text {q}>1\%$ in both strain fields – deviatoric and volumetric (the white dashed line defines the boundaries “engaged volume” used to plot $\varepsilon _\text {q}$ vs. $\varepsilon _\text {v}$). The plots on the right hand-side show volumetric strain $\varepsilon _v$ versus deviatoric strain $\varepsilon _q$. From top to bottom: the effect of maize root growth on fine denser sand, fine looser sand, and coarse looser sand.

Growing plant roots not only invade existing pores, but they also create their own space by displacing soil particles (Supplementary Figure S6), which disturbs the soil matrix^32^. Here, such disturbance is assessed by 3D Digital Image Correlation (DIC)^33^ between a reference configuration – Day 0 – and a deformed one – the rest of the chronosequence. A discrete DIC approach is adopted for the coarse Hostun sand: each individual sand particle is detected, the position of its centre of mass is calculated and tracked in space and time to measure particle displacements. A local DIC approach is used for small fixed-size cubic sub-volumes of fine Hostun sand, called correlation windows. Here, the total specimen volume containing fine sand is divided into a structured grid of about 158401 correlation windows, with a side length of 1.3 mm. Displacements and strains are computed using the open source software SPAM – Software for Practical Analysis of Materials^34^ – at each measurement point (centre of mass for coarse sand and centre of correlation window for fine sand). Concerning the fine sand, local strains are calculated from the displacements obtained from image correlation in the finite strain framework, using a 3D implementation of the method proposed by Geers et al.^35^. The first invariant – the volumetric strain $\varepsilon _\text {v}$ – is calculated as the determinant of the transformation gradient (**F** − **I**). The second invariant, which represents the maximum shear deformation – the deviatoric strain $\varepsilon _\text {q}$ – is calculated as the Euclidean norm of the deviatoric part of the strain tensor. For the coarser sand, a tetrahedral mesh is obtained by a Delaunay triangulation^36^, and discrete strain is computed according to Bagi’s formulation^37^.

Figure 7 compares 2D slices of both fields in the three soil configurations under study. In the figure $\varepsilon _q$ is displayed as the projection of the maximum shear strain in the plane of observation, and $\varepsilon _v$ is displayed as the average volumetric strain of measurement points adjacent to the plane of observation. The deviatoric strain field reveals that the root shears the soil while growing, in both sands and for both densities. The root-induced shear strain decreases as the distance to the root surface increases (see the colour maps in Fig. 7). Correspondingly, the volume of soil engaged by root growth is defined as the volume in which the shear strain exceeds a threshold strain $\varepsilon _q > 1\%$. The choice of this threshold, represented by a white dashed line in Fig. 7 and explained in detail in Anselmucci et al.^38^, allows filtering of the noise in the shear strain calculations.

**Figure 8:**
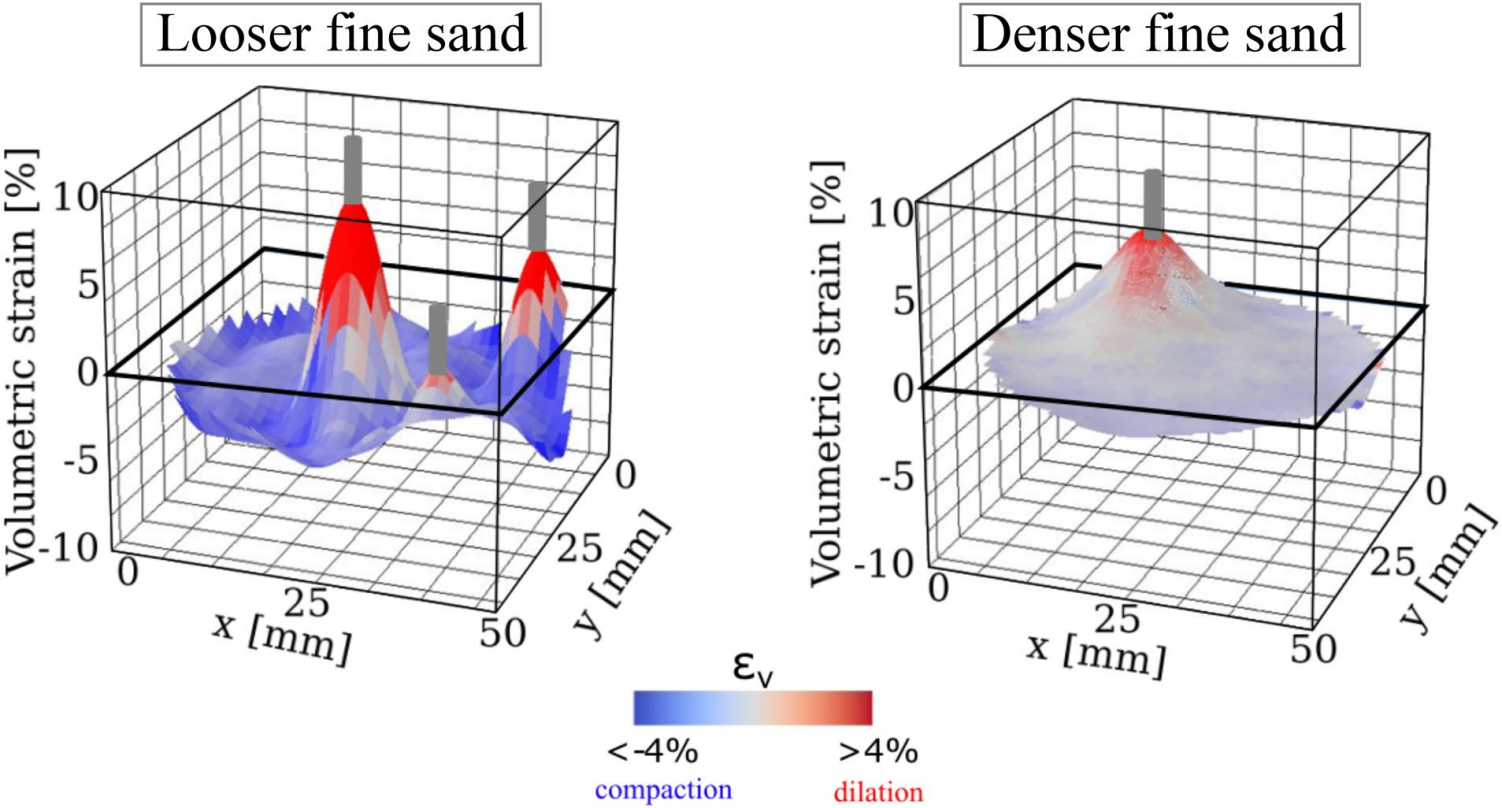
3D surface plots of volumetric strain in the vicinity of the root axis in looser (left) and denser (right) sand. The data correspond to the root system at Day 3 at depth of 37.7–39 mm. The soil experiences dilation (red) close to the root for both densities. The soil experiences compaction further from the root for the looser sand only.

In the following, the strain fields are analysed in the “engaged volume” (i.e., where $\varepsilon _q > 1\%$). For the highest values of $\varepsilon _q$, the engaged soil exhibits dilation (red pixels), in the three configurations. This is well illustrated in the plots of volumetric strain versus deviatoric strain in Fig. 7, which also highlights a clear difference between looser and denser configurations: in looser sand, the centroid of the cloud of points at low deviatoric strain (represented by the orange line) is in the negative domain (indicating compaction), while in the denser soil, the volumetric strain is in average dilative at small deviatoric strain ($\varepsilon _\text {q}<3\%$). This result implies that further from the root, the soil dilates in denser sand, while it compacts in looser sand^38^. Figure 8 further illustrates this phenomenon, by showing a hyper-surface of volumetric strain in the vicinity of the root axis for one particular horizontal slice at Day 3 at depth of 37.7–39 mm.

**Figure 9:**
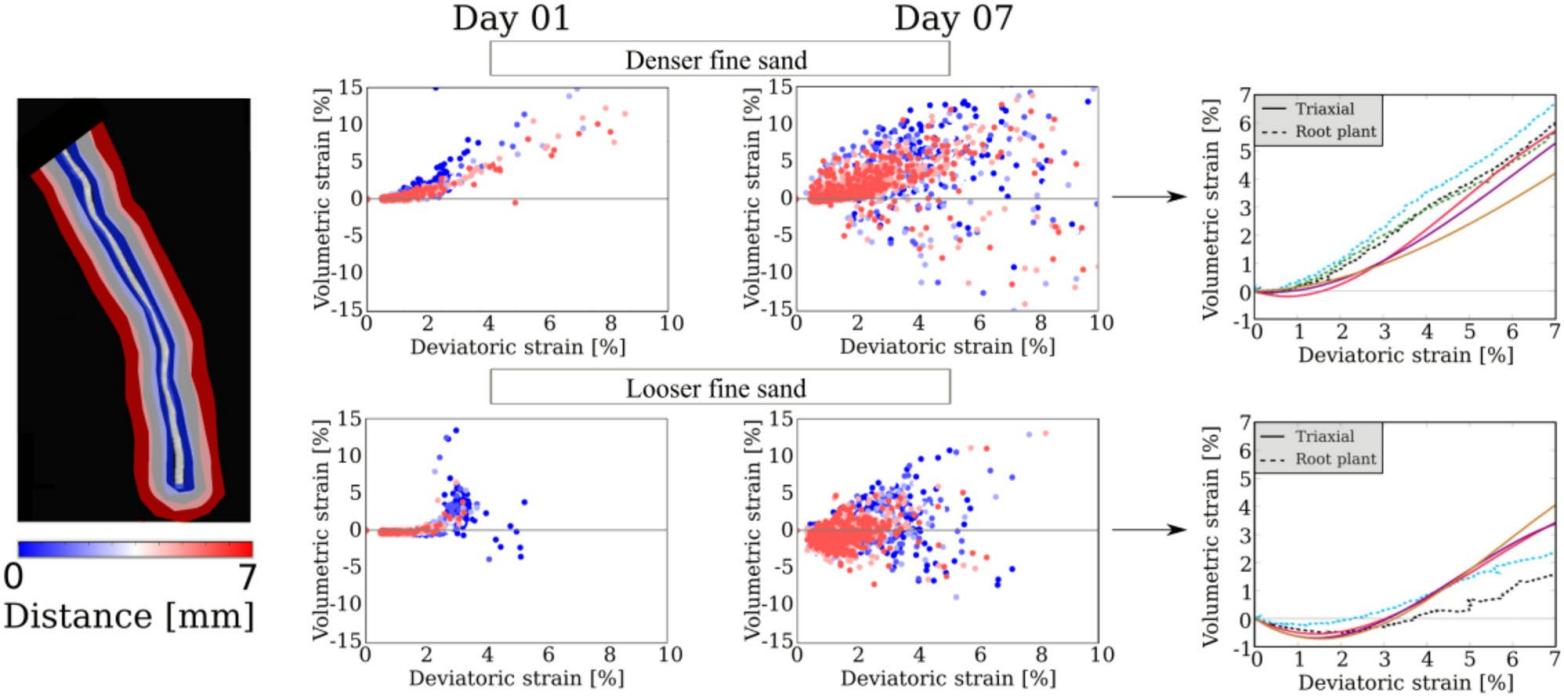
Plots of volumetric strain versus deviatoric strain in the vicinity of the primary root at Day 1 and Day 7. Blue dots (respectively, red dots) refer to points located in the immediate vicinity (respectively, further neighbourhood) of the root, i.e., in the blue zone (respectively, red zone) in the left picture. On the right hand-side, the interpolation line of the clusters (continuous line) are compared to strains measured during compression triaxial tests^27^ (dashed lines).

**Figure 10:**
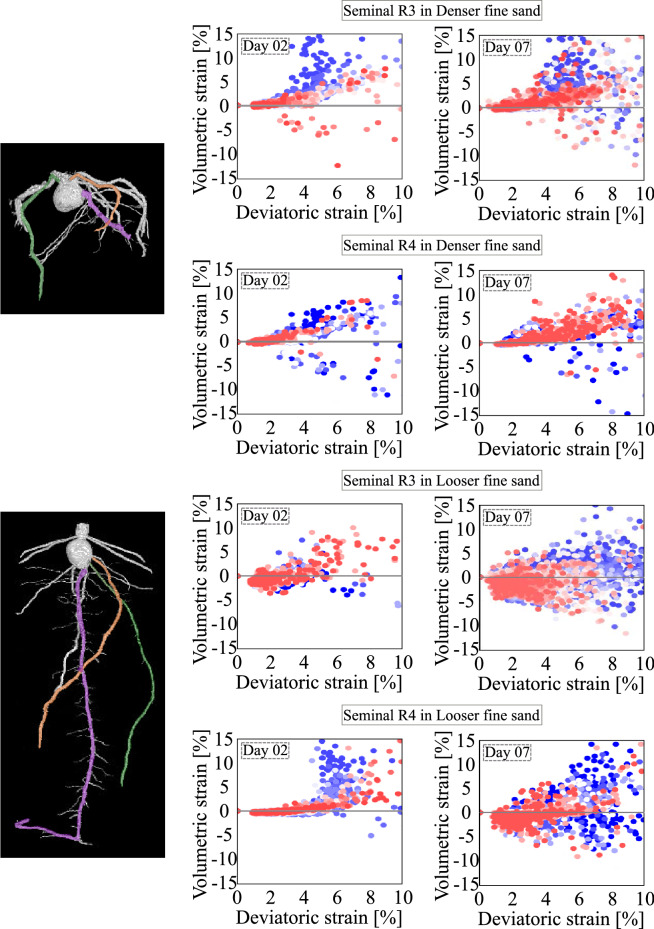
Plots of volumetric strain versus deviatoric strain in the vicinity of the seminal roots R3 (green) and R4 (orange) on Day 2 and Day 7. Blue dots (respectively, red dots) refer to points located in the immediate vicinity (respectively, further neighbourhood) of the roots. For reference, the primary root (R1) is highlighted in purple.

In order to better understand the effect of the moving root tips, strains are analysed in the immediate vicinity of a single root axis (the primary root). To do this, the coordinates of the correlation windows that contain portions of the primary root are identified, and the strains of the portion of soil surrounding the specific root are extracted. Figure 9 shows the results for two representative days (i.e., Day 01 and Day 07). From the plots displayed in Fig. 9, it is clear that, for smaller values of deviatoric strain, the position of the centroid of the cloud of points representing the volumetric deformation depends on the initial relative density of the soil: the growth of the primary root shears the soil, which induces dilation close to the root (red dots), and either dilation or compaction further away (for initially denser and looser soil, respectively, see the blue dots). This analysis is repeated in Fig. 10 for two other seminal roots, on Days 2 and 7. The results are very close to those obtained with the primary roots, leading to identical conclusions.

Results of triaxial compression tests conducted at very low confining pressure (i.e., 5 kPa) on Hostun sand HN31 – previously called Hostun RF^14,16^ – at different initial relative densities^27^ are compared to the strain variations observed around the primary root (Fig. 9, right hand-side). This comparison shows that the behaviour induced by root shearing is not unusual and that the strain variations observed in the triaxial tests are similar to those induced by root growth, for both densities. This observation could guide the choice of constitutive models for prediction of the soil response to root-induced shear at low confining pressure.

## Summary and closing remarks

The presented work provides new data concerning the mechanical response of sand to the natural growth of plant roots. The specific protocol designed to reproduce the early stages of maize root growth at laboratory scale reveals that the cross-section thickness of the roots and the root length density do not depend on the initial soil configuration. The data obtained from X-ray computed tomography are processed to extract measurements on both soil kinematics and root system architecture. The analysis on the root system architecture indicates that the initial density of the soil is the parameter that affects the most the final architecture. This is mostly confirmed by the depth reached by the root tips and the number of second-order laterals. In coarser sand, the lower capillary rise hinders access to water, which limits especially the development of lateral roots. The growth of the maize roots increases the sand porosity in the vicinity of the root and the dilated zone characteristic length depends on the initial density of the soil, about $30.0\,\times D_{50}$ and $7.4\,\times D_{50}$ for the denser and looser configuration, respectively. The accuracy of this results are in line with the conclusions already obtained from literature.

The novelty of this work lies in the quantification of the soil strain tensor by image analysis during plant root growth (in vivo). It was found that while elongating, roots shear the sand, which translates in a dilation of the sand in the vicinity of the root body. Initial density affects the response of the sand further away from the root, where denser sand experience low-magnitude dilation, whereas looser sand experience compaction. This behaviour is also observed in sand subjected to compression under a few kPa of confining pressure. Hence, two sharply divergent patterns have emerged from the strain analysis far from the root. However, a common dilatant response is found immediately around the root. Therefore, on the basis of these results, we may assume that the porosity fall-off trends described in this paper and in prior studies are likely to be correlated to the shear induced by the root elongation, and not only from steric exclusion.

The results of this study have important implications on how to optimise plant growth and yield in agriculture. Literally, an overly stressed soil will not be conducive to productive crops. Additionally, the root-induced deformation mechanisms highlighted in this study have the potential to inspire the design of new geotechnical probes meant to penetrate the soil and to assess soil local fabric and stress from local deformation fields. One of the major challenges in validating the root architecture deployment behaviours is the difficulty to control or assess the water content distribution during in-vivo experiments. Additionally, a smaller incremental step of observation will give more specific information on phenomena still under investigation, such as the link between root branching chronosequence and soil features. Such results could be obtained by fast-neutron tomography^39–41^. These open issues are left for future work.

## Supplementary information

Supplementary Information.
