# Supplementary material for: Imaging local soil kinematics during the first days of maize root growth in sand
Source: Sci Rep. 2021 Nov 15;11:22262. doi: 10.1038/s41598-021-01056-1 (PMC8593153; doi:10.1038/s41598-021-01056-1)
Supplement: Supplementary file 1 — Supplementary Information. [file 41598_2021_1056_MOESM1_ESM.pdf]

Imaging local soil kinematics during the first days of maize root growth in sand

Supplementary Information

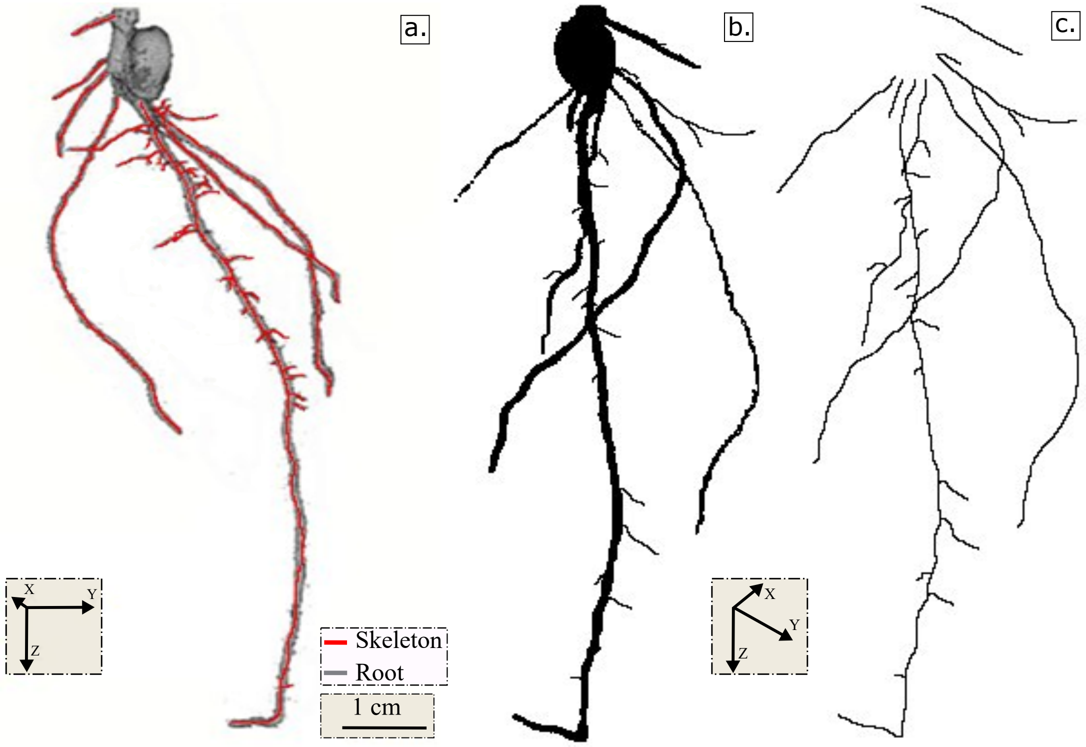

Figure S1: Example of skeleton extraction using the python module `skimage.morphology.skeletonise3D`. 4-day old Maize root system growth in the looser fine sand (in grey the root system, in red the corresponding skeleton) (**a.**). The same Maize root system, 6-day old, from a different angle (**b.**), and its skeleton (**c.**).

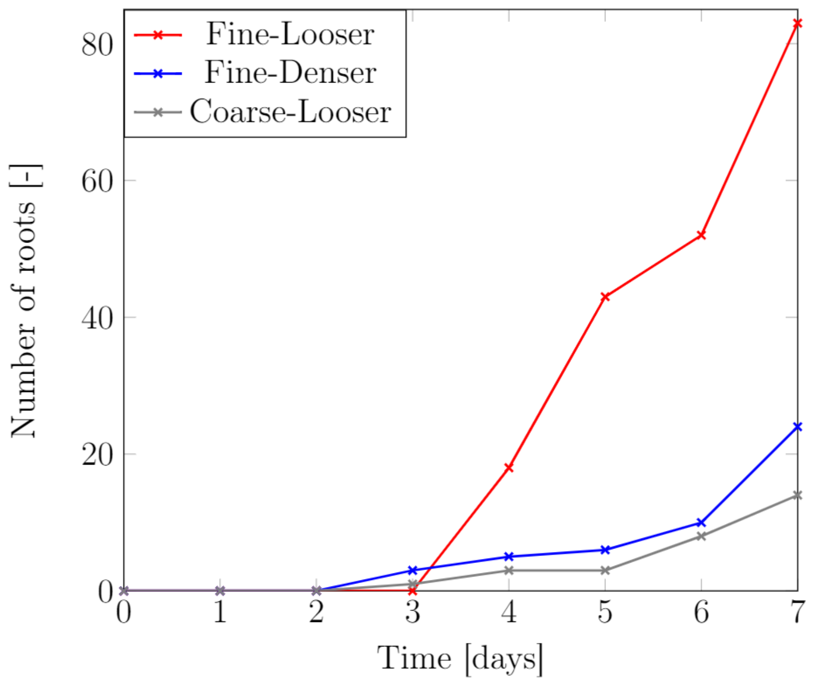

Figure S2: Time evolution of the number of maize second order lateral roots in the three different soil configurations studied in this work. The 7-day old seedling growth in the looser fine sand develops the highest number of second order laterals.

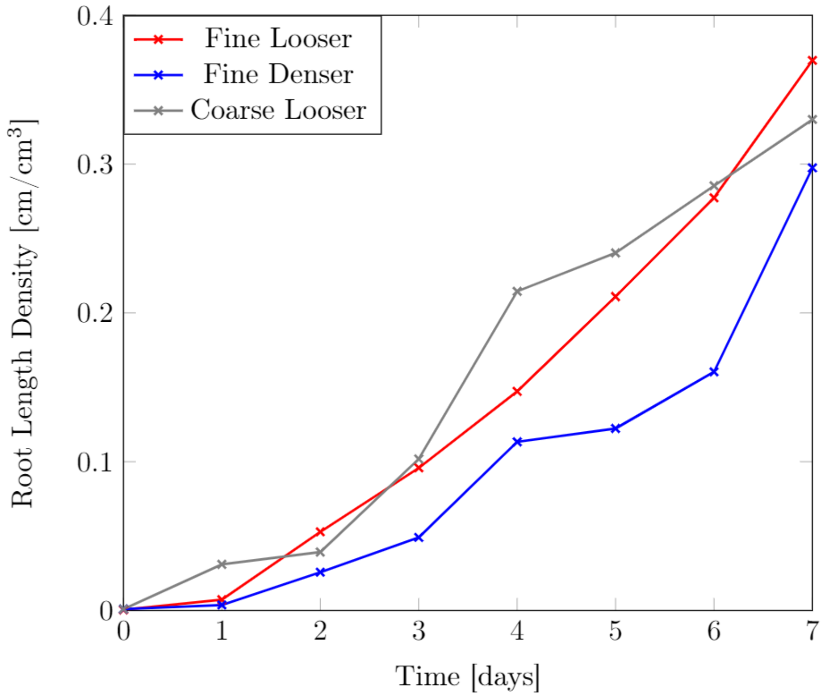

Figure S3: Time history of the root length density for the maize root systems in each of the three soil configurations.

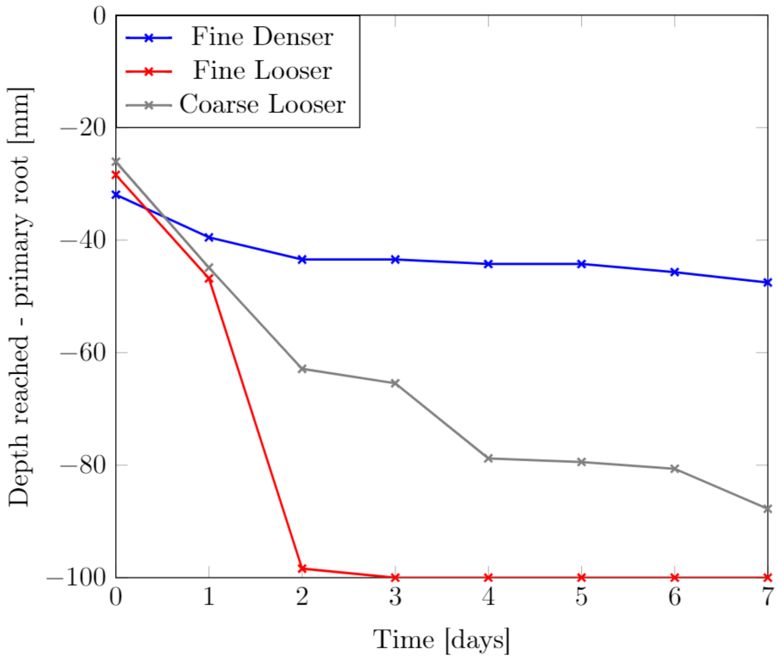

Figure S4: Time series of the depth reached by each maize primary root in the three soil configurations. The root system in the looser fine sand reaches the bottom of the cell on Day 3, while in the finer dense after Day 2, the elongation rate is almost null. In the coarser looser sand the root elongation progressively increases in time, reaching almost the bottom of the container on Day 7.

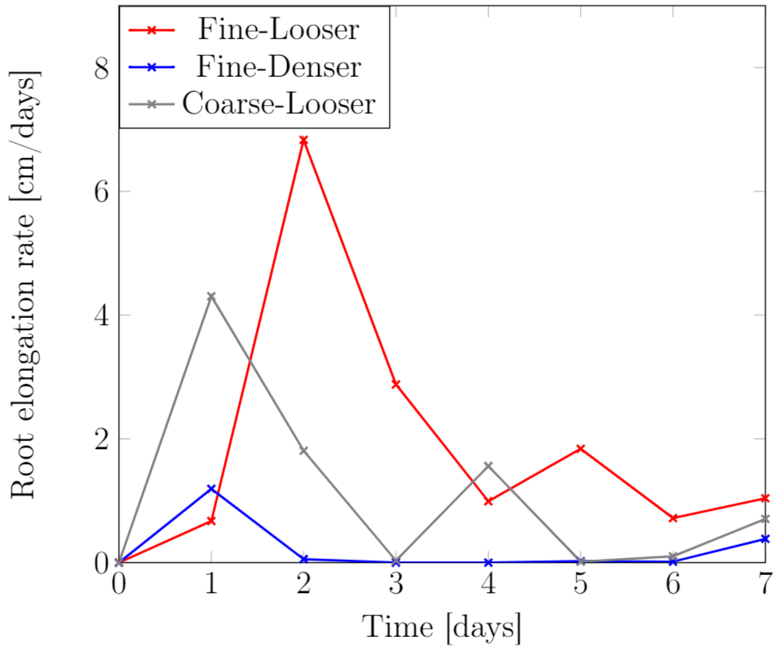

Figure S5: Root elongation rate of the primary root for the three different soil configurations.

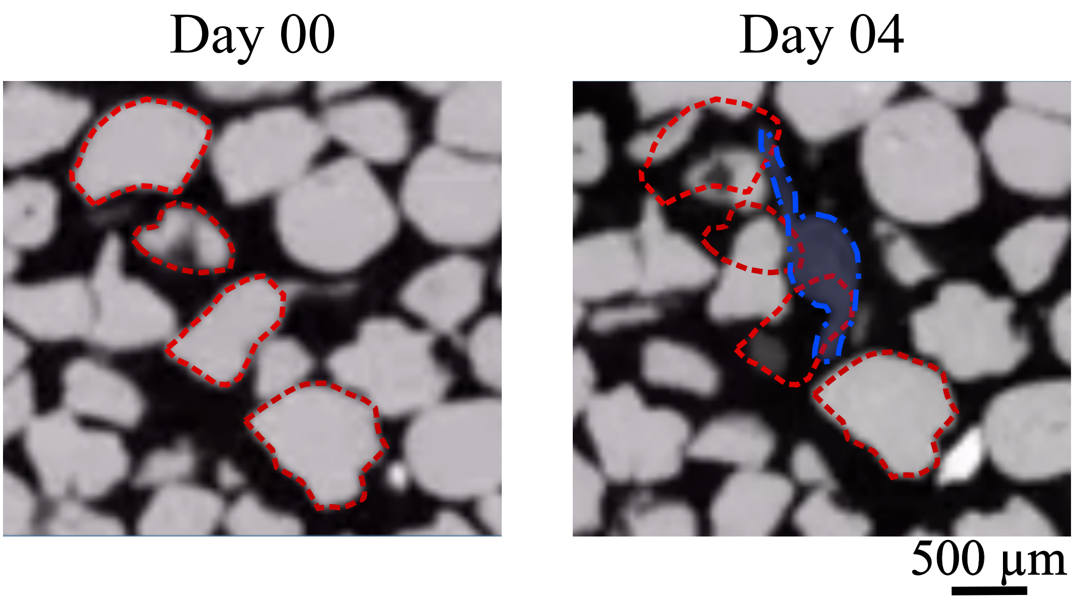

Figure S6: Raw data showing the displacement of 4 grains (red dashed line) due to the maize root growth (blue dashed line), after 4 days. The root has approximately the same size as the pores, but it does not fill the pre-existing pores and displaces the sand particles, as illustrated in this figure.

Properties	
Voltage	135kV
Current	220mA
Spot size	Middle
Source filter	0.2 mm Cu
Pixel size	40 μm
Number of projections	2240
Number of reference projections	32
Detector orientation	Portrait
Number of tomography stacks	2
Average frame	10
Scan duration	1h50

Table S7: Settings of the x-ray scanner of Laboratoire 3SR in Grenoble (France) used to obtain the time-series of the root-soil systems.

	**Fine Denser**				**Fine Looser**				**Coarse Looser**			
**Properties**	xr	nxr-1	nxr-2	nxr-3	xr	nxr-1	nxr-2	nxr-3	xr	nxr-1	nxr-2	nxr-3
Stem length [cm]	6.2	7.1	6.7	6.3	10.3	11.6	10.9	10.3	7.5	8.1	10.3	8.2
Number of leaves	4	5	4	4	6	5	6	6	5	5	6	5
Max leave length [cm]	15.3	15.3	14.4	16.7	20	22.3	18.6	19.5	15.8	15.3	18.3	19.2
Primary root length [cm]	8.7	10.3	5.6	7.9	22.3	17.7	21.2	20.5	13.7	15.3	14.9	11.5
Number of 1st order roots	11	12	10	9	14	15	16	14	10	10	8	9

Table S8: Evaluation of X radiation effects on maize plant development after 18 days of growth. Properties of x-rayed sample (xr) are compared to those of 3 non-x-rayed (nxr) samples, for each of the three soil configurations.

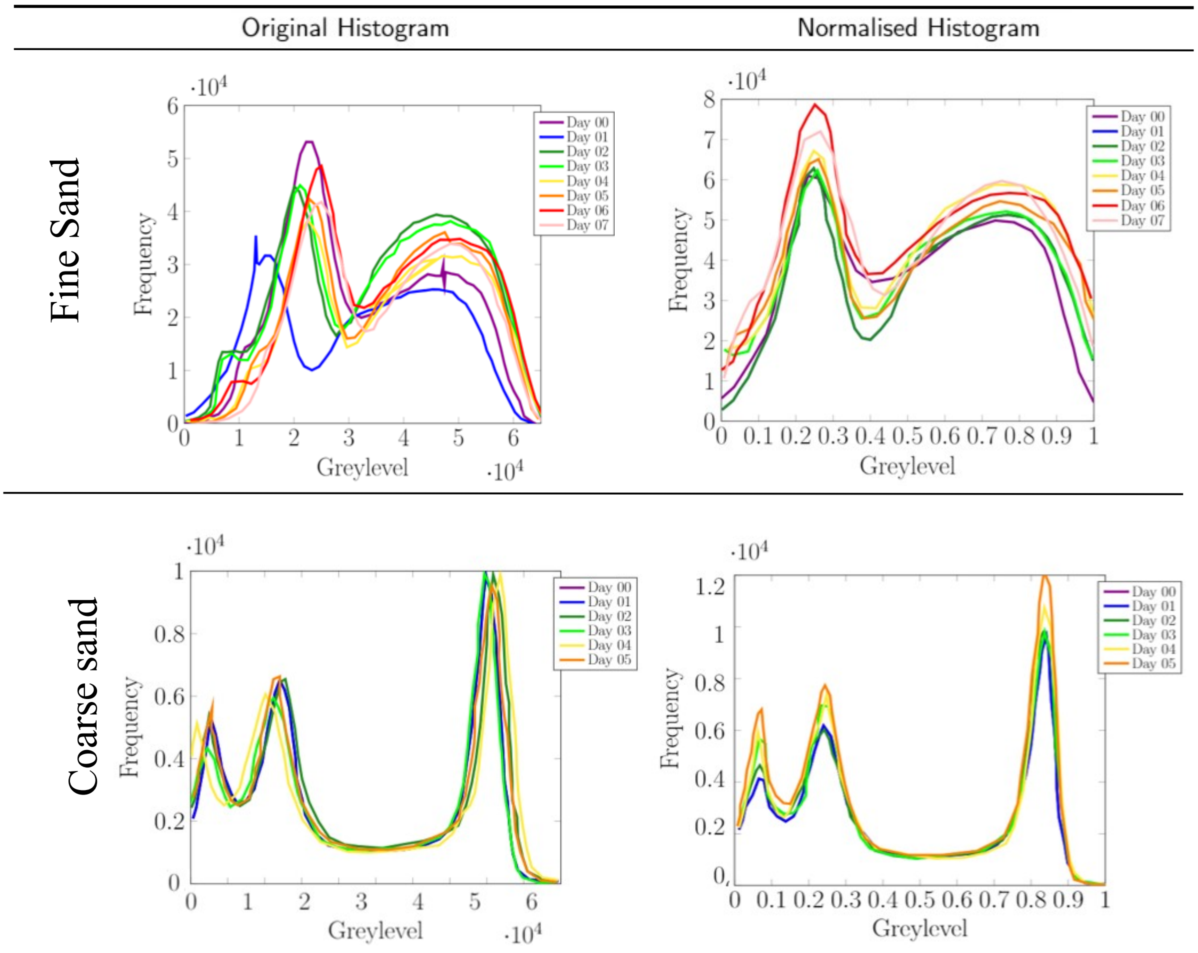

Table S9: Examples of the greyvalue distributions of the sets of 3D images of the root-soil systems. On the left, original distributions. On the right, after linearisation of the grey value of each voxel with respect to the greyvalues at the peaks of the distributions. Distribution peaks are representative of the main phases of the system, the frequency at each peak may change according to the volume occupied by the corresponding phase; but the greyvalue at the peak, typical of the density of the material constituting the phase, is assumed to remain constant during the 7 days of observation.
